# pH-Responsive Metal–Organic Framework Thin Film for Drug Delivery

**DOI:** 10.1021/acs.langmuir.2c02497

**Published:** 2022-12-14

**Authors:** Steven G. Guillen, Jacob Parres-Gold, Angel Ruiz, Ethan Lucsik, Benjamin Dao, Tran K. L. Hang, Megan Chang, Adaly O. Garcia, Yixian Wang, Fangyuan Tian

**Affiliations:** † Department of Chemistry and Biochemistry, California State University Long Beach, 1250 Bellflower Boulevard, Long Beach, California90840, United States; ‡ Department of Chemistry and Biochemistry, California State University Los Angeles, 5151 State University Drive, Los Angeles, California90032, United States

## Abstract

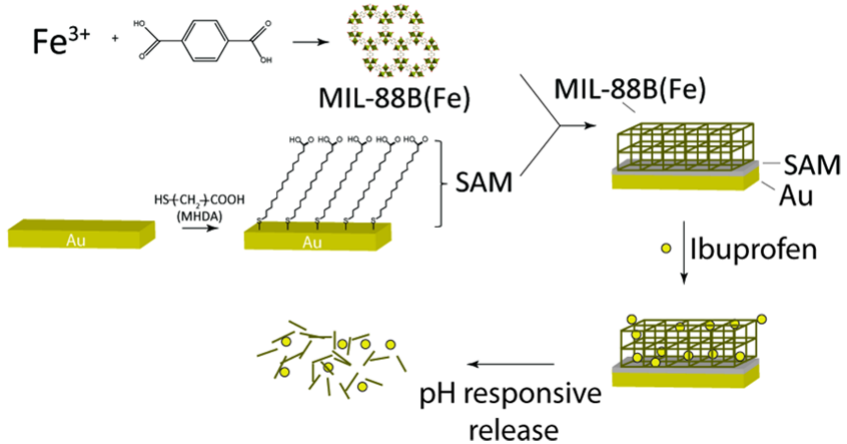

In this work, surface-supportive MIL-88B(Fe) was explored as a pH-stimuli thin film to release ibuprofen as a model drug. We used surface plasmon resonance microscopy to study the pH-responsive behaviors of MIL-88B(Fe) film in real time. A dissociation constant of (6.10 ± 0.86) × 10^–3^ s^–1^ was measured for the MIL-88B(Fe) film in an acidic condition (pH 6.3), which is about 10 times higher than the dissociation of the same film in a neutral pH condition. MIL-88B(Fe) films are also capable of loading around 6.0 μg/cm^2^ of ibuprofen, which was measured using a quartz crystal microbalance (QCM). Drug release profiles were compared in both acidic and neutral pH conditions (pH 6.3 and 7.4) using a QCM cell to model the drug release in healthy body systems and those containing inflammatory tissues or cancerous tumors. It was found that the amount of drug released in acidic environments had been significantly higher compared to that in a neutral system within 55 h of testing time. The pH-sensitive chemical bond breaking between Fe^3+^ and the carboxylate ligands is the leading cause of drug release in acidic conditions. This work exhibits the potential of using MOF thin films as pH-triggered drug delivery systems.

## 1 Introduction

The development of pH-responsive drug delivery systems has been continuously investigated throughout the past decade. An increased emphasis has been placed on pH gradients and their significant contributions as environmental stimuli for the triggering of drug release events occurring within human organs, tissues, and at the cellular level. For instance, the gastrointestinal (GI) tract contains a broad pH range between 1 and 7.5, which results in various retention times of therapeutic compounds within the body when they are orally administered.^1^ Also, tumor cells tend toward having a slightly acidic extracellular environment (pH 6.0–7.0) when compared to healthy tissues (pH 7.2–7.4).^2^ All of these concerns have made the design of pH-responsive drug carriers an urgent need. Compared to conventional drug delivery systems in response to pH stimuli, including polymeric micelles, liposomes, polyplexes, and silica nanoparticles, there is still room for the improvement of drug loading amounts and pharmacokinetics.^3−8^ Therefore, we call our attention to a type of organic–inorganic hybrid materials, metal–organic frameworks (MOFs), which have become highly touted in contemporary biomedical applications on account of their sizable pore volumes, high surface areas, biocompatibility, and their pH-related chemical stabilities.^9−13^

MOFs, also referred as crystalline coordination frameworks, are unique hybrid materials formed by the self-assembly of metal ions/clusters to selective binding organic ligands. Their development originated from molecular crystal engineering in the 1990s.^14,15^ The focus of using MOFs has brought upon the assembly of organic and inorganic building blocks to form novel two-dimensional (2D) and three-dimensional (3D) porous structures for biomedical applications.^16−18^ Compared with traditional drug carriers, MOFs hold the characteristics of both organic and inorganic systems: similar to polymeric materials, MOFs are flexible and chemically tunable;^19,20^ meanwhile, certain MOFs are stable as inorganic drug carriers. The presence and the characteristics of the metal sites in MOFs that hold together the organic linkers are to a considerable extent responsible for the structural and compositional complexity of a given MOF.^21^ More importantly, selective MOFs and their composites exhibit stimuli-responsive behaviors toward endogenous and exogenous stimuli when being applied as drug delivery systems.^8,22^ For instance, zeolitic imidazolate framework-8 (ZIF-8), composed of Zn^2+^ and 2-methylimidazole (a pH-sensitive group), was used for intracellular delivery of DNA polymerase and nucleic acid probes when the pH drops below 6.^23^ Another type of MOF-based material, polydopamine-functionalized MIL-53(Fe), was reported to be loaded with an antitumor drug, camptothecin, with pH-sensitive drug release profiles.^24^

To expand the use of bulk materials, surface-supportive MOFs (SURMOFs) have been explored as applications for electronic devices, sensors, and medical purposes. As a promising drug delivery system, a SURMOF layer can function as a topical treatment to release therapeutic components in a controlled manner. Selective SURMOF thin films also have the potential to replace nondegradable polymers in drug-eluting stents to release drugs for treating atherosclerosis.^25^ There are two typical approaches to building SURMOFs: physisorption and chemisorption. For a stronger binding, we focus on the latter, where a thin organic layer is fashioned using techniques such as electropolymerization, plasma polymerization, or self-assembled mono- and multilayers.^26^ In this work, we selected a COOH-terminated self-assembled monolayer (SAM) to connect an iron-containing MOF, MIL-88B(Fe) (MIL stands for Material from Institute Lavoisier), on top of gold surfaces. MIL-88B(Fe) is composed of Fe trimmers connected with terephthalate ligands by forming a three-dimensional porous structure.^27,28^ The resulting cages are flexible and can compensate for the size of guest molecules at certain degrees, which is known as the “breathing effect”.^30^ The low toxicity of Fe-based MIL materials was confirmed by both *in vitro* and *in vivo* toxicological studies.^31,32^ The safety and structural flexibility have promoted MIL-88B(Fe) to be used for drug delivery.^10,29^

However, applying SURMOF thin films for drug delivery requires further investigation to hone its effectiveness. There are several challenges that need to be addressed: (1) uniformity of the MOF thin film; (2) binding between SURMOF films and the surface; and (3) film degradation after drug release. Fabrication of SURMOF films by wet chemistry is usually achieved through these two approaches: direct growth/deposition from a solvothermal method using pretreated crystallization solution (often referred to as the mother solution) and layer-by-layer (LBL) growth by exposing the target surface with metal clusters and ligands in a stepwise manner.^33^ The use of a SAM in the direct growth of SURMOF enables the strong binding between the MOF layer and the substrate; meanwhile, it is easy to control the surface coverage and uniformity.^34^ The first observation of SAMs for direct growth of zeolites subsequently led to the pioneering study utilizing MOF (MOF-5) on SAM-modified Au surfaces in a supersaturated mother solution reported by Hermes et al.^35^ In this study, they found that grafting SAMs allowed for precise control over the growth of MOF-5 on surfaces similar to zeolite thin films, thus spawning revolutionary ideas for tailoring chemical and physical functionality at the molecular level. Another study by Biemmi et al. reported the first tunable and oriented crystal growth at the molecular level of another well-known MOF, HKUST-1, on different functionalized SAMs on Au (−COOH and −OH termini).^36^ This concept was furthered in a study by Scherb et al.^37^ In our previous study, MIL-88B(Fe) was fabricated on a COOH-terminated SAM.^38^ The XRD patterns provided evidence that our crystals oriented exclusively in the [001] direction, implying that the MIL-88B(Fe) crystals grew in parallel to the Au surface, as determined by the coordination of the carboxylate coordinated metal clusters of the MOF. Ultimately, the carboxylate functionality from the 16-mercaptohexadecanoic acid (MHDA) SAM simulates the carboxylate groups of the 1,4-benzenedicarboxylate (bdc) ligands in MOF, resulting in oriented growth.^37−39^

Characterizing the assembly process of MOF is crucial for understanding the process at the molecular level as well as for elucidating the importance of secondary building units and for studying the growth of selective MOFs on various substrates. Surface adsorption and desorption behaviors can be studied in real time with the aid of analytical techniques, for example, surface plasmon resonance (SPR) and quartz crystal microbalance (QCM). Both techniques have been used to study the growth mechanisms of selective MOFs.^40−43^ In the present work, we utilize a state-of-the-art SPR microscopy (SPRm) method to determine the desorption kinetics of a surface-supportive MIL-88B(Fe) film at various pH conditions. SPRm bolsters the standard SPR technique by integrating an optical microscope concurrently during SPR detection.^44,45^ The sensor uses a light condenser which illuminates the sample in tandem with an optical microscope camera that captures the bright field images of the mounted sensing chip. Simultaneously, the SPR projects a light beam at a given resonance angle which alters the propagation of the surface plasmon oscillating waves at the shared interface. Ultimately, these waves are reflected and captured by the SPRm detector after being scattered by a surface-bound object. SPRm images the intensity change, which reflects the local refractive index change that can be associated with chemical and biological reactions.^46,47^ In this work, we also use QCM to study pH-dependent drug-releasing behaviors of the resulting film as a function of time. QCM is designed based on the piezoelectric effect that the quartz probe oscillates when an alternating current is applied. The oscillation frequency of the quartz sensor is associated with the mass atop the quartz crystal. For a rigid thin film, the frequency change and the mass increase/decrease caused by surface adsorption/desorption can be described by the Sauerbrey equation:^48^
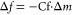
1where Δ*m* is the change in mass per unit area in μg/cm^2^, Δ*f* is the observed frequency change in Hz, and Cf is the sensitivity factor for the crystal (56.6 Hz μg^–1^·cm^2^ for a 5 MHz AT-cut quartz crystal at room temperature). Given that the crystal structure of both MHDA SAM and MIL-88B(Fe) film are rigid, we were able to directly apply the Sauerbrey equation to estimate the surface desorption related to the material degradation and drug release.

## 2 Experimental Section

### 2.1 Chemicals and Materials

Chemicals used include terephthalic acid (Acros Organic 99+%), hydrogen peroxide (Fisher Chemical, 30%), dimethylformamide (DMF, Fisher Chemical, 99.9%), ibuprofen (Acros Organic, 99%), iron chloride hexahydrate (FeCl_3_·6H_2_O, Acros Organic, 99+%), Milli-Q water (Milli-Pore, 18.2 MΩ·cm), ammonia hydroxide (Millipore Sigma, 30%), and phosphate-buffered saline (PBS, Gibco, pH 7.2, 1×, with 0.5% Tween20). All chemicals were reagent grade or better and used as received.

AT-cut piezoelectric quartz crystal sensors coated with Au with a resonant frequency of 5 MHz (Stanford Research System) as well as gold-coated silicon wafers (50 ± 5 nm of Au on 500 ± 30 μm p-type Si (111), Ted Pella) were also used in this study.

### 2.2 Sample Preparation

#### 2.2.1 Synthesis of MIL-88B(Fe)

MIL-88B(Fe) was synthesized according to previously reported procedures with some modifications.^27,49^ Terephthalic acid (0.116 g, 1 mmol) and FeCl_3_·6H_2_O (0.270 g, 1 mmol) were dissolved in 5 mL of DMF in a glass reactor, followed by the addition of 0.4 mL of 2.0 M NaOH and sonicating for 2 min. The sonicated solution was decanted into separate reaction vials (6 dram glass vials), all placed in an oven at 100 °C for 12 h. The resulting mother solution was run through vacuum filtration to separate its MIL-88B(Fe) bulk powder component. The mother solution was further centrifuged (5000 rpm for 15 min) to separate smaller MIL-88B(Fe) solids from DMF. The separated MIL-88B(Fe) then underwent a solvent exchange by resuspension in 200 proof ethanol followed by three cycles of centrifugation. From these steps, there were two distinct substances created: a MIL-88B(Fe)/ethanol solution (mother solution) and bulk MIL-88B(Fe) powder. MIL-88B(Fe) thin films were made using the mother solution, and the bulk powder that can be collected from previous vacuum filtrations was then later used for XRD and IR studies. When using the bulk powder, it was first thoroughly washed using DI water and acetone and then dried overnight in an oven at 110 °C before characterization.

#### 2.2.2 Preparation of Functionalized Au

Prior to any experimentation, the gold-coated quartz crystal sensors and gold-coated silicon wafers were thoroughly cleaned using a standardized procedure: The gold-coated wafers were sonicated for 5 min in ethanol before being placed in a UV-ozone cleaner (Bioforce Nanosciences) for 10 min. Next, they were subjected to a preheated (75 °C) 5:1:1 ratio of Milli-Q water, 30% ammonia solution, and hydrogen peroxide for 5 min, followed by thorough rinsing with Milli-Q water. Rinsed samples were then dried with nitrogen gas before being placed in the UV-ozone cleaner for 10 min. Lastly, the precleaned gold-coated wafers were then immersed in an MHDA-ethanol solution (1 mM) for 24 h at room temperature to grow a COOH-terminated SAM.

#### 2.2.3 Preparation of SURMOF MIL-88B Thin Films

An MHDA-functionalized gold-coated substrate was placed in the centrifuged MIL-88B(Fe)/ethanol mother solution and incubated for 24 h at room temperature within an enclosed glass container with its gold-plated side facing upward. After direct crystallization soaking, the sample was rinsed with ethanol before being dried with nitrogen gas.

### 2.3 Drug Delivery Studies

#### 2.3.1 Drug Loading into a MIL-88B(Fe)-Modified Gold Surface

Following the MHDA/MIL-88B(Fe) functionalization of the gold-coated surface, the substrate was then incubated in a freshly prepared 0.5 mg/mL of ibuprofen in hexane solution in a shaker for 24 h. The sample was then rinsed thoroughly with fresh hexane and ethanol, in sequence, to remove any residual surface adsorbed ibuprofen before drying with nitrogen gas. These final samples were the standardized functionalized Au chips prepared for surface characterization and further drug release experiments. Any subsequent drug loading amounts were then determined by measuring discrete changes in resonance frequency (*f*, in Hz) of the sensor using a quartz crystal microbalance (QCM). For clarity, the final fully functionalized gold-plated wafers (herein referred to as fully functionalized gold samples) progressed through the following surface modification steps: (i) MHDA/ethanol, (ii) MIL-88B(Fe)/ethanol, and (iii) ibuprofen/hexane.

#### 2.3.2 Drug Release Studies

A fully functionalized gold sample loaded with ibuprofen was immersed in 60 mL of PBS with 0.5% Tween 20 at room temperature across varying lengths of time. Simultaneously, the QCM was used to continuously monitor for discrete changes in the raw frequency of the sample *in situ*.

### 2.4 Characterization Techniques

#### 2.4.1 Attenuated Total Reflectance Infrared Spectroscopy (ATR-IR)

A Fourier transform infrared spectrometer (FTIR) with an attenuated total reflectance accessory was used to obtain attenuated total reflectance infrared (ATR-IR) spectra. The spectra were collected at a resolution of 16 cm^–1^ and 128 scans per measurement in a range of 4000–400 cm^–1^. Air was used as the background.

#### 2.4.2 X-ray Diffractometer (XRD)

Powder X-ray diffraction analysis was performed using a Bruker D2 Phaser with a Cu Kα radiation source. The acquisitions were carried out in the 2θ range of 5 to 40 degrees with a step size of 0.02 degrees. Powder MIL-88B(Fe) was loaded into a PMMA ring holder for XRD analysis.

#### 2.4.3 Scanning Electron Microscopy (SEM)

SEM images were obtained on a Phenom ProX G6 system (Thermo Fisher). The images were taken by a backscattered electron detector with an acceleration voltage of 15 kV in vacuum conditions. Prior to imaging, all samples were sputter-coated with a thin layer of gold and palladium to increase conductivity for better resolution.

#### 2.4.4 Quartz Crystal Microbalance (QCM)

A QCM200 system (SRS, Inc.) attached with a QCM25 crystal oscillator for (1″ diameter 5 MHz AT-cut crystals) was used to collect real-time frequency data for baseline open-air experiments. All baseline readings were allowed to oscillate for a minimum of 10 min prior to measurements for requisite equilibration. A desired gold-coated quartz crystal was secured in the crystal head holder with a retainer cover. Data was collected for a minimum of 24 h or until discernible fluctuations in frequency were no longer apparent. Any prominent change in frequency, and thus any change in mass, either positive or negative, of the gold sample following each modification step, as well as the drug-releasing properties of the MIL-88B(Fe) film, were verified by analysis of the raw frequency data in conjunction with the Sauerbrey relation, as described in eq 1.

#### 2.4.5 Surface Plasmon Resonance Microscopy (SPRm)

Gold SPRm sensing chips were functionalized as previously noted, cleaned with ethanol, and dried with nitrogen gas prior to use. A Flexi Perm silicon well (Sarstedt) was also cleaned in triplicate using ethanol and Milli-Q water and then dried with nitrogen gas. The silicon well was then centered atop the fully functionalized gold sensing chip by gently pressing.

For flow cell studies, an enclosed flow cell attachment was placed atop the functionalized gold sensing chip, and PBS solution (0.05% Tween 20) was flowed at 196 μL/min with varying pH levels. The pH levels used ranged from 6.3 to 7.8 and were previously adjusted accordingly using monobasic and dibasic sodium phosphate solution. All PBS solutions were also filtered using Stericup Quick Release Filtration Systems (0.22 μm pore size). Data collection was completed using Image SPR software (Biosensing Instruments). Prior to each sample collection, an angle sweep was completed immediately following the addition of the PBS solution. An angle sweep was calibrated and assigned on the left side at approximately 3/4 of the resulting SPR curve (i.e., a dip). All samples were allowed to run to completion until a plateau reading was reached.

For each sample completed, all resulting raw SPRm image stack files were first converted to tif stack files using ImageAnalysis (Biosensing Instruments), extracted using Fiji (ImageJ2, Version 2.3.0/1.53f) to .tiff sequences, and then processed with MATLAB (R2021b, Version 9.11.1769968) to produce all subsequent disassociation plots, histograms, and heatmaps for determining kinetic parameters.

## 3 Results and Discussion

### 3.1 pH-Sensitive Surface-Supportive MIL-88B(Fe) Thin Films

**Scheme 1:**
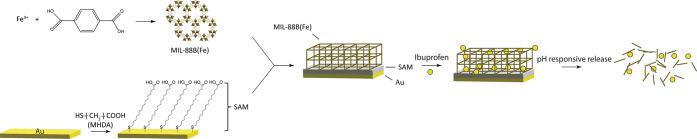
Schematic Illustration of Surface Modification Steps in This Study: Preparation of MIL-88B(Fe) Film on MHDA SAM-Functionalized Au Surface, Ibuprofen Drug Loading, and pH-Responsive Drug Release Behaviors Ibuprofen is represented by solid yellow circles. The thickness of each layer is not scaled.

**Figure 1:**
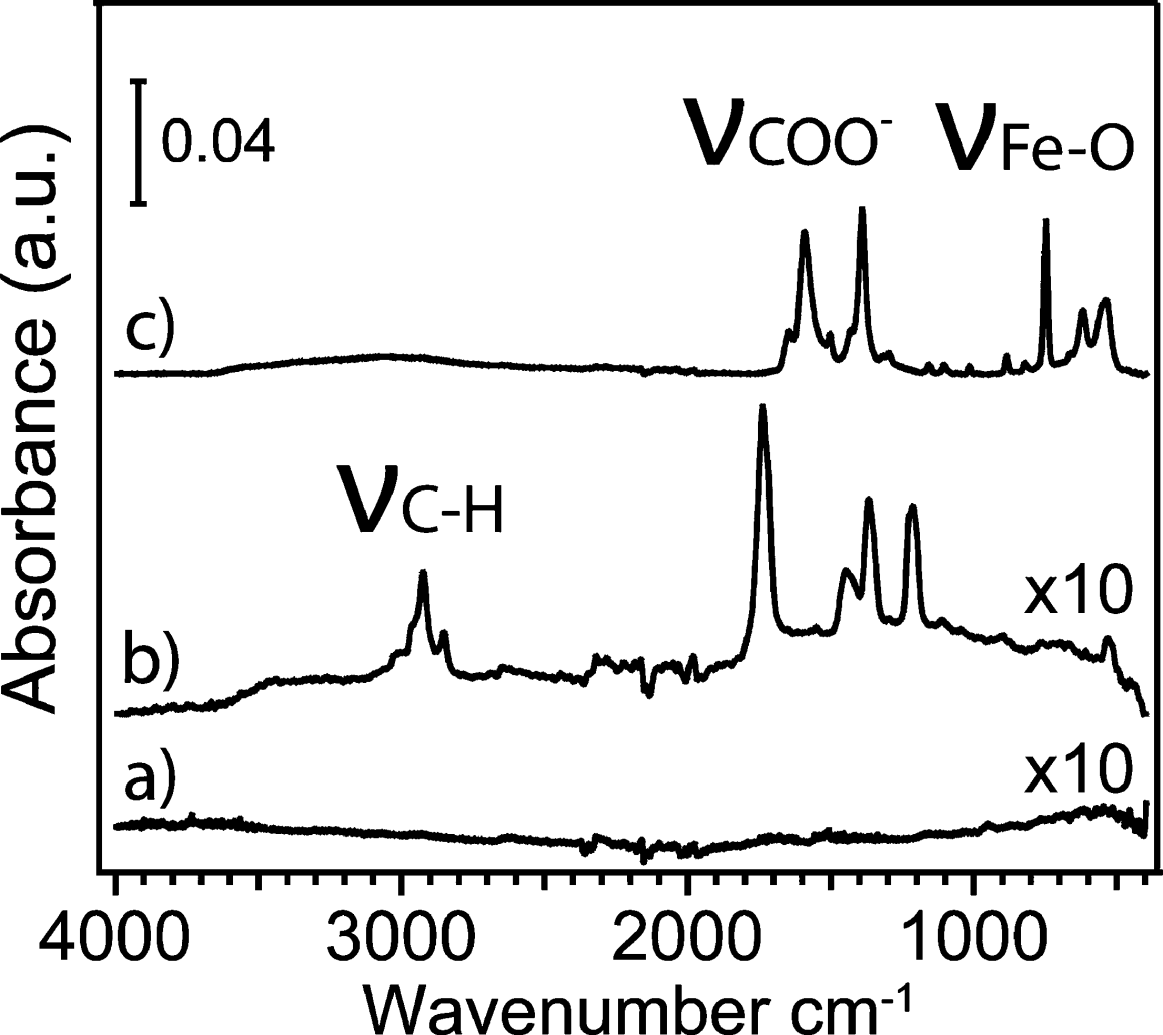
ATR-IR spectra of (a) clean Au, (b) MHDA SAM-modified Au, and (c) MIL-88B(Fe)-coated MHDA SAM-functionalized Au surfaces.

**Figure 2:**
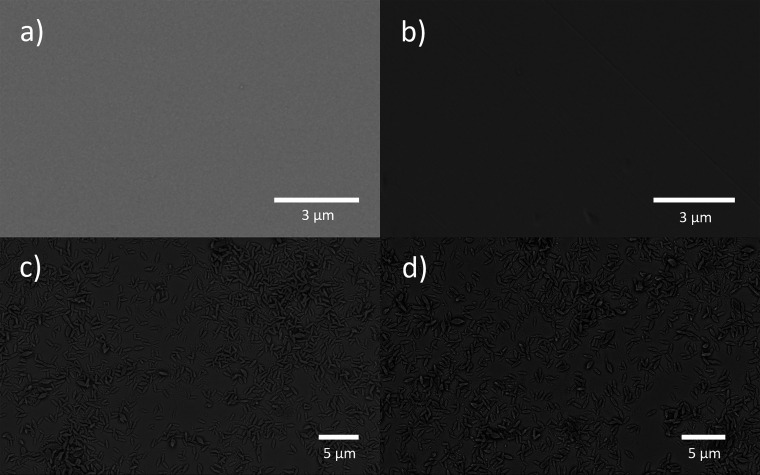
SEM images of (a) a clean Au chip, (b) MHDA SAM-modified Au, (c) MIL-88B(Fe)-coated MHDA SAM-functionalized Au, and (d) ibuprofen-loaded MIL-88B(Fe)-coated MHDA SAM-functionalized Au surfaces.

MIL-88B(Fe) crystals were produced through a solvothermal method by heating the synthesis solution to 100 °C for 12 h. Both MIL-53(Fe) and MIL-88B(Fe) can be prepared from the same starting materials (terephthalic acid and ferric chloride hexahydrate). MIL-53(Fe) is formed through homogeneous nucleation at a higher temperature (150 °C), while MIL-88B(Fe) is resulted from heterogeneous nucleation.^31,37^ The powder X-ray diffraction (PXRD) result, Figure S1, was found to be in excellent agreement with previously reported data on MIL-88B(Fe) bulk crystal^27,28^ and confirmed the successful synthesis of pure MIL-88B(Fe). A scanning electron microscopic (SEM) image, shown in Figure S2, demonstrated the long rice grain-shaped MIL-88B(Fe) crystals; the morphology of the synthesized MIL-88B(Fe) was also consistent with previously reported results.^50^ We then used the resulting mother solution to fabricate MIL-88B(Fe) thin film on MHDA-SAM-modified Au surfaces. The complete surface modification process is illustrated in Scheme 1. The film formation was confirmed by our ATR-IR analysis after each modification step, as shown in Figure 1. The C–H stretch features were observed at 2856 and 2928 cm^–1^, respectively, on the surface of the MHDA-SAM-modified Au. After the surface was modified with MIL-88B(Fe), a characteristic Fe–O stretch was observed at 543 cm^–1^. The symmetric and asymmetric stretching of the carboxylate groups were noticed at 1391 and 1594 cm^–1^, indicating a successful attachment of MIL-88B(Fe) on the SAM-modified Au surface. We also performed SEM studies to monitor the surface morphology changes after each modification step. As shown in Figure 2, the Au surface was flat after cleaning and the modification of MHDA SAM. A layer of MIL-88B(Fe) crystals with the characteristic “rice-grain” shapes were noticed after the thin film preparation, shown in Figure 2c. Both IR and SEM studies confirm the formation of MIL-88B(Fe) on the functionalized Au surface.

**Figure 3:**
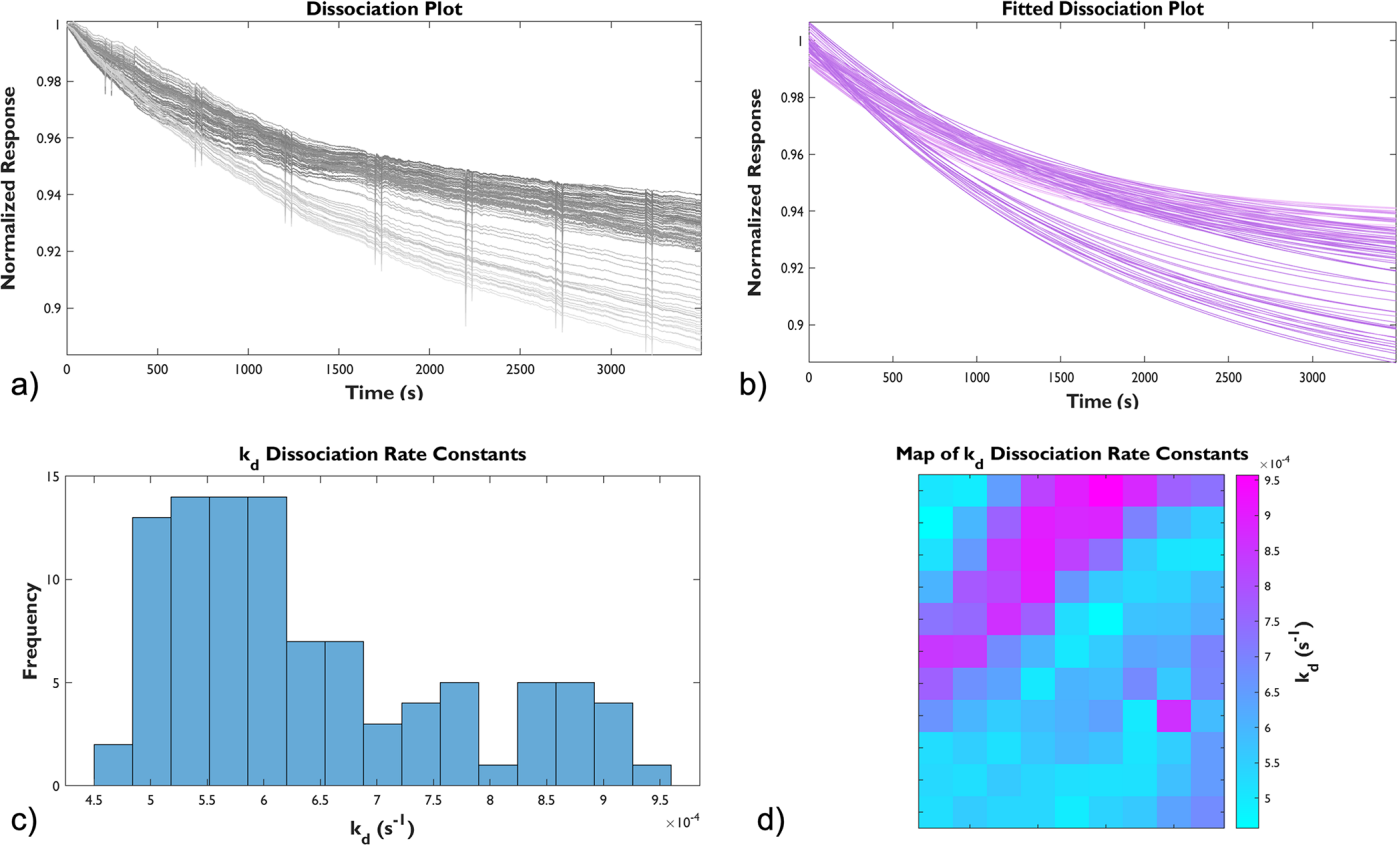
Responsiveness of MIL-88B(Fe) film on a functionalized SPRm Au sensing chip at pH 7.2. (a) Raw dissociation plots and (b) the fitted dissociation plots from 99 ROIs. (c) The histogram resulting from the fitted dissociation constants, *k*_*d*_, from all ROIs. d) A 2D gradient heatmap representing the distribution of *k*_*d*_ at the sensing chip surface (600 × 450 μm).

**Figure 4:**
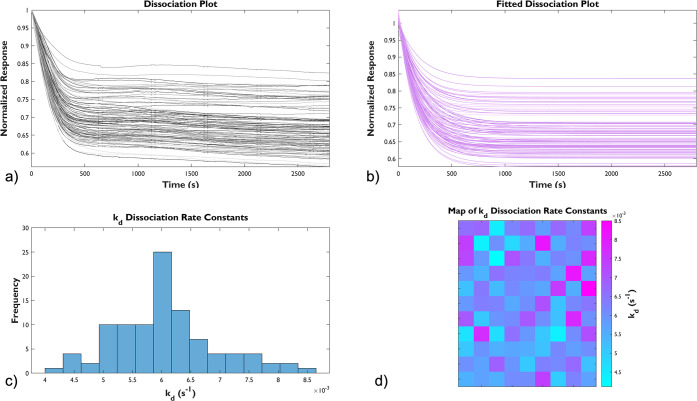
Responsiveness of MIL-88B(Fe) film on a functionalized SPRm Au sensing chip at pH 6.3. (a) Raw dissociation plots and (b) the fitted dissociation plots from 99 ROIs. (c) The histogram resulting from the fitted dissociation constants, *k*_*d*_, from all ROIs. (d) A 2D gradient heatmap representing the distribution of *k*_*d*_ at the sensing chip surface (600 × 450 μm).

**Figure 5:**
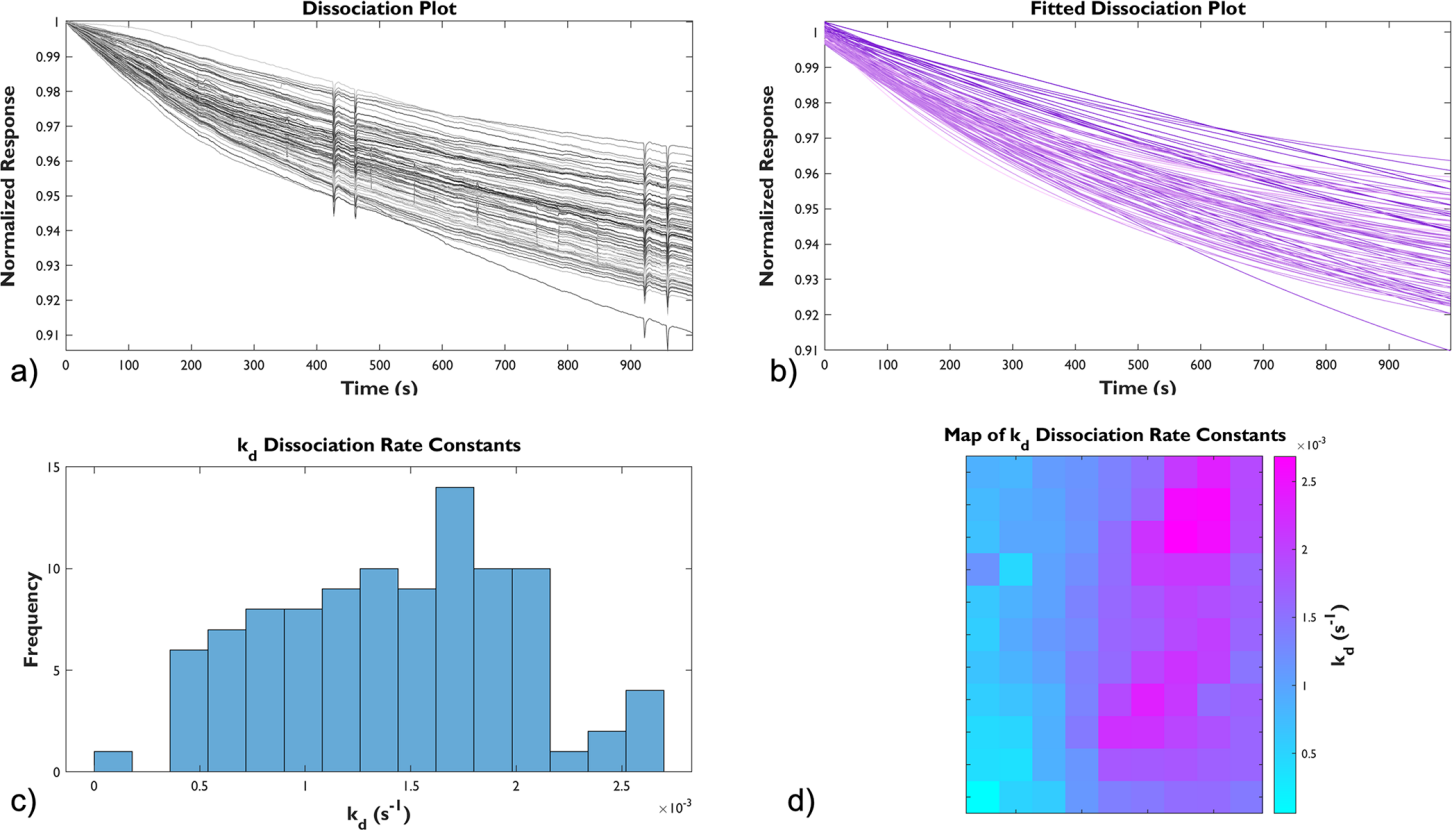
Responsiveness of MIL-88B(Fe) film on a functionalized SPRm Au sensing chip at pH 7.8. (a) Raw dissociation plots and (b) the fitted dissociation plots from 99 ROIs. (c) The histogram resulting from the fitted dissociation constants, *k*_*d*_, from all ROIs. (d) A 2D gradient heatmap representing the distribution of *k*_*d*_ at the sensing chip surface (600 × 450 μm).

Following confirmation of the formation of MIL-88B(Fe) film on the functionalized substrate, we utilized SPRm for a better understanding and cataloging of the pH-responsiveness of the resulting MIL-88B(Fe) film. Our work was directed toward understanding the nuances of how MIL-88B(Fe) thin films would respond as they were introduced to flowed PBS solution of varying pH levels (pH 7.2, 6.3, and 7.8), with an emphasis on monitoring the kinetic details of dissociation of our MOF during the initial one hour. During SPRm analysis, the scanned area (600 × 450 μm) was divided into 99 regions of interest (ROIs), and the real-time dissociation response was monitored from each individual ROI. A general dissociation trend characterized by an exponential decay was observed in all three pH levels, as shown in Figures 3–5. Raw dissociation plots from all ROIs (panel a in Figures 3–5) were normalized by the starting signal and fitted to an exponential decay described by the following model,
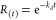
2where *R*_(*t*)_ is the normalized measured SPRm response and *k*_*d*_ is the dissociation constant. Theoretical fitted plots are shown in panel b of Figures 3–5. *k*_*d*_ values extracted from all successful fittings are plotted as histograms (panel c of Figures 3–5) and statistically analyzed, from which the mean, median, and standard deviation are listed in Table 1. SPRm also allows visualization of the distribution of *k*_*d*_ values over the entire sensing area, which is presented by heatmaps in panel d of Figures 3–5. The heatmaps provided the localized data of all the graphs as each “pixel” (i.e., a colored square) represented by the unique kinetic information (*k*_*d*_) of each ROI.

**Table 1:** Summary of MIL-88B(Fe) Film Dissociation Constants under Various pH Conditions

pH	Mean *k*_*d*_ (s^–1^)	Median *k*_*d*_ (s^–1^)	STD (s^–1^)
6.3	6.10 × 10^–3^	6.10 × 10^–3^	8.60 × 10^–4^
7.2	6.39 × 10^–4^	6.00 × 10^–4^	1.27 × 10^–4^
7.8	1.40 × 10^–3^	1.50 × 10^–3^	5.80 × 10^–4^

Figure 3, a neutral condition (pH 7.2), served as a representative baseline to give context to the acidic and basic conditions which followed. A general trend in dissociation was observed throughout the entirety of the analysis in Figure 3a. In similarity, there was an observed general trend in dissociation in Figure 4a; but, with clear distinction, a sharply defined dissociation (drop to 60∼80% of starting signal) was observed within the initial 300 s of the trial under the acidic condition (pH 6.3). Responses under the slightly basic condition (pH 7.8) were more similar to the neutral condition. The same observation was noted in Figure 5a, albeit to a much lesser extent. The observed dissociation spanned over a markedly longer time period and was much less pronounced under basic conditions. Statistical analysis shows that the dissociation under the acidic condition is almost 10 times faster than that under the neutral condition ((6.10 ± 0.86) × 10^–3^ s^–1^ vs (6.39 ± 1.27) × 10^–4^ s^–1^), and the dissociation under the basic condition ((1.40 ± 0.58) × 10^–3^ s^–1^) is in between the previous two conditions; all these data were summarized in Table 1. Lastly, the degradation of MIL-88B(Fe) at various pH levels was visualized on heatmaps associated with its corresponding dissociation constants, also indicating a faster decomposition of MIL-88B(Fe) film in the acidic environment, consistent with the previously reported aqueous stability of bulk MIL-88B(Fe).^13^ We concluded that surface-supportive MIL-88B(Fe) degraded faster under either acidic or basic conditions compared to those in neutral. The innate low stability of MIL-88B(Fe) films in acidic or basic conditions can be advantageously exploited for applications to characterize their use for pH-responsive drug delivery. It is worth noting that the degraded products of MIL-88B(Fe), including terephthalic acid and Fe^3+^ ions, exhibit no adverse effects on human with a LD_50_ value of more than 5000 mg/kg for terephthalic acid^51^ and a recommended daily intake amount of 18 mg for an adult female.^52^ Therefore, it is safe to consider using MIL-88B(Fe) in drug delivery applications.

### 3.2 pH-Responsive Drug Delivery by MIL-88B(Fe) Thin Films

To investigate the possibility of using MIL-88B(Fe) thin film as a pH-responsive drug carrier, we used QCM to evaluate drug loading amounts and to monitor drug release at various pH conditions in real time. QCM is more feasible for longer time analysis of surface adsorption/desorption process *in situ* compared to SPRm.^40^ Surface-supportive MIL-88B(Fe) was prepared on MHDA SAM-modified QCM sensors, followed by loading with ibuprofen as previously described.^38^ We chose ibuprofen as a model drug due to the following reasons: (1) ibuprofen is an over-the-counter nonsteroidal anti-inflammatory drug (NSAID) that is commonly used for easing pain; (2) the size of the ibuprofen molecule (4 × 6 × 10 Å) is comparable to the entrance aperture of MIL-88B(Fe) cages (9.5 × 19.0 Å),^28^ allowing for spontaneous drug loading; (3) ibuprofen together with the cages of MIL-88B(Fe) are hydrophobic, which promotes drug encapsulation, and (4) ibuprofen is stable in PBS at various pH conditions. Our previous studies on powder MIL-88B(Fe) for delivery of ibuprofen have demonstrated that the drug release is associated with three factors: drug diffusion, surface, and bulk erosion of the carrier.^31^ More specifically, drug molecules diffusing from the cages and surface of MIL-88B(Fe) can be the leading cause of drug release when it is first immersed in PBS. Then, MIL-88B(Fe) starts hydrolysis by breaking down to smaller pieces and dissolving in PBS, which leads to more drug release. Our previous HPLC (high performance liquid chromatography) studies show the accumulative ibuprofen release amount increases with the increasing concentration of terephthalic acid (the building block of MIL-88B(Fe)), indicating a linear relationship between drug release and the decomposition of MIL-88B(Fe).^31^ In this study, we monitored the change in resonance frequency of the MIL-88B(Fe)-modified sensor chip before and after ibuprofen loading using a QCM. Since the MIL-88B(Fe) film together with the SAM beneath it are considered rigid, the Sauerbrey equation (eq 1) can be used to convert the frequency change to a change in mass. We observed a mass increase of 6.0 ± 4.0 μg/cm^2^ over the span of six trials of ibuprofen being loaded on surface-supportive MIL-88B(Fe) film. The large standard deviation was due to the variable amounts of MIL-88B(Fe) attached to the MHDA SAM-modified QCM sensors. Based on our SEM studies shown in Figure 2c,d, the MIL-88B(Fe) film remains intact after ibuprofen loading.

**Figure 6:**
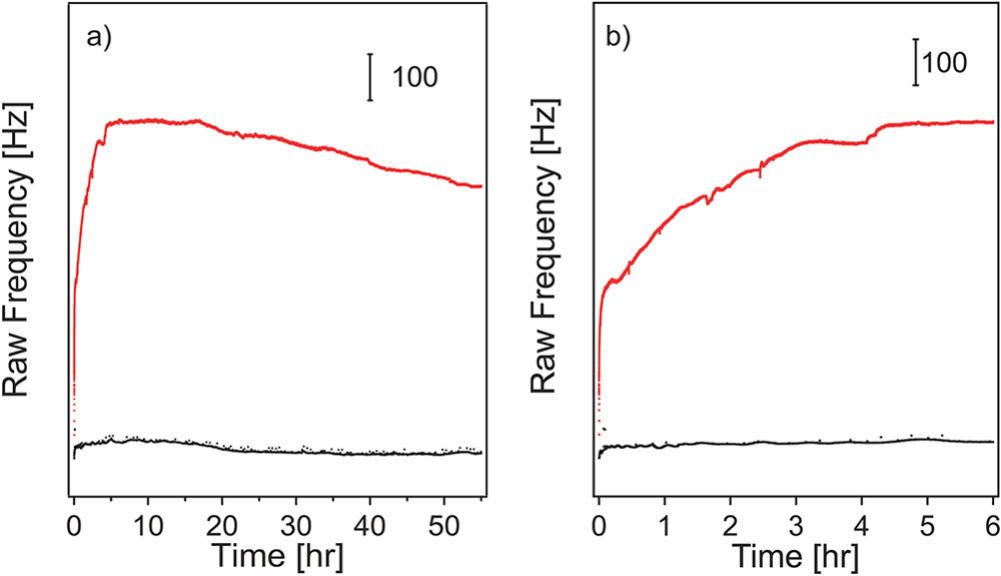
Real-time QCM measurements of ibuprofen released by MIL-88B(Fe) film on MHDA-modified Au sensor chips in PBS at pH of 6.3 (red) and pH of 7.4 (black). (b) The zoom-in region for the first 6 h in both cases.

**Figure 7:**
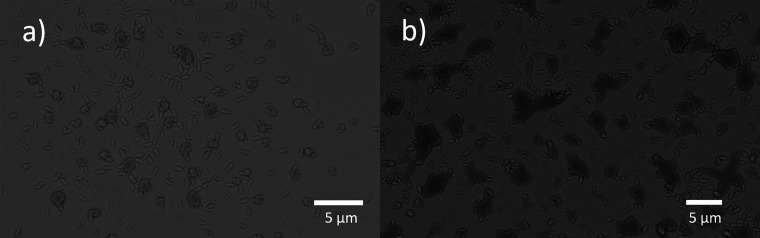
SEM images of MIL-88B(Fe)-coated MHDA SAM-functionalized Au surfaces after 55 h of ibuprofen release in PBS at (a) pH = 7.4 and (b) pH = 6.3.

To compare the drug-releasing process at different pH levels *in situ* using QCM, we ensured that all tested sensor chips were with a similar amount of ibuprofen loaded into the MIL-88B(Fe) films. Here, all tested QCM chips were functionalized with MHDA and MIL-88B(Fe) and were then incubated in the ibuprofen/hexane solution in a shaker for a minimum of 24 h. After that, the chip was rinsed thoroughly with hexane to remove any surface adsorbed ibuprofen, followed by rinsing with ethanol to remove hexane. The chip was dried with nitrogen gas before being placed in the QCM probe to perform a dry measurement to confirm the drug loading amount. We selected the QCM chips loaded with about 8 μg/cm^2^ ibuprofen for the following drug release experiments. A drug-loaded QCM probe head was immersed vertically in PBS at a certain pH level, and the resonance frequency was monitored for up to 55 h. Figure 6 presents the frequency changes of ibuprofen-loaded MIL-88B sensors as a function of time in PBS at pH of 7.4 and 6.3, respectively. As shown in Figure 6a, the resonance frequency did not change much (11.0 Hz) for the sensor in the PBS at pH 7.4, corresponding to an overall of 0.2 μg/cm^2^ of surface desorption after 55 h of immersion calculated based on the Sauerbrey equation (eq 1). In contrast, the frequency increased dramatically for the chip immersed in the PBS at pH 6.3. During the first 6 h, Figure 6b, a raw frequency change of 680 Hz was observed, corresponding to 12.0 ug/cm^2^ of mass decrease on the MIL-88B(Fe) film-coated Au surface at pH 6.3, indicating a burst delivery of ibuprofen in an acidic environment. The overall desorbed mass was higher than the drug loading amount because some MIL-88B(Fe) crystal degraded in the acidic PBS, which also contributed to the mass change, and QCM is sensitive to any mass change of the sensor chip. We also noticed that both curves show an increase in frequency in the first 15 h then followed by a slight decrease in frequency over time. This is presumably due to the readsorption of ibuprofen and some MIL-88B(Fe) crystals during the static immersion process. By comparing the surface desorption phenomena at two pH levels, the MIL-88B(Fe) film released more ibuprofen in a much more rapid manner in the tested acidic condition. We believe this is due to the pH-sensitive coordination bond between iron and terephthalate ligands. In an acidic condition, hydrogen ions behave as Lewis acids and will compete with metal ions to bind with organic ligands (Lewis base), thus leading to a faster bond breakage between iron and terephthalate and further facilitating the ibuprofen release. This explanation was also confirmed by our SEM studies on surface morphology of MIL-88B(Fe) film after drug release at two different pH conditions. We noticed that both films degraded after such a long period of soaking in PBS, as shown in Figure 7. However, the film treated at pH of 7.4 still showed a good amount of regular shaped MIL-88B(Fe) crystals on the surface (Figure 7a), whereas the surface treated at pH of 6.3 exhibited more surface defects with irregular shaped MIL-88B(Fe) crystals left (Figure 7b), indicating more material degradation occurred during drug release at a lower pH, which is consistent with our QCM results. Additionally, we compared the drug release profiles of MIL-88B(Fe) film with its bulk format which was carried out in our previous study.^31^ The trends for ibuprofen release at neutral pH for both MIL-88B(Fe) film and powder are similar. According to prior literature, MIL-88B(Fe) tends to be far less stable during extended soaking in aqueous and at lower or higher pH conditions,^13,50^ which could explain the different drug release behaviors of MIL-88B(Fe) film at pH of 7.4 and 6.3. Overall, by comparing our SPRm, QCM, and SEM results, we believe the drug release is due to ibuprofen diffusion and the decomposition of MIL-88B(Fe) framework, and the process of degradation is accelerated at lower pH conditions, making the material pH-responsive for drug delivery.

## 4 Conclusions

In this study, we explored the pH-responsiveness of MIL-88B(Fe) using the SPRm method to describe its kinetic details under a variety of pH conditions, including acidic, neutral, and basic environments. This work provided a set of uniquely localized kinetic information of surface-supported MIL-88B(Fe) film in real time. Beyond this, our study has demonstrated a new approach to study the degradation of surface-supportive MOF thin films using SPRm. The dissociation constant of MIL-88B(Fe) in acidic conditions is about one magnitude higher than that in neutral conditions; thus, MIL-88B(Fe) is considered as a pH sensitive material. Moreover, we investigated the drug-loading and -releasing capabilities of MIL-88B(Fe) at varying pH conditions *in situ* by employing a static immersion QCM method. Our combined SPRm and QCM studies confirmed the potential use of MIL-88B(Fe) as carriers for pH-responsive drug release applications. This work will also support future research to find optimal experimental conditions for loading and unloading of guest molecules using other surface-supportive MOF films. Most importantly, the knowledge gained from these studies will advance the development of other pH-responsive drug delivery systems using organic–inorganic hybrid materials.

## Supporting Information Available

PXRD pattern and SEM image of MIL-88B(Fe) powder (PDF)

## References

[ref1] Russell T. L.; Berardi R. R.; Barnett J. L.; Dermentzoglou L. C.; Jarvenpaa K. M.; Schmaltz S. P.; Dressman J. B. Upper Gastrointestinal PH in Seventy-Nine Healthy, Elderly, North American Men and Women. Pharm. Res. 1993, 10 (2), 187–196. 10.1023/A:1018970323716. 8456064

[ref2] Vaupel P. Tumor Microenvironmental Physiology and Its Implications for Radiation Oncology. Semin. Radiat. Oncol. 2004, 14 (3), 198–206. 10.1016/j.semradonc.2004.04.008. 15254862

[ref3] Gao W.; Chan J. M.; Farokhzad O. C. pH-Responsive Nanoparticles for Drug Delivery. Mol. Pharmaceutics 2010, 7 (6), 1913–1920. 10.1021/mp100253e. PMC3379544 20836539

[ref4] Kocak G.; Tuncer C.; Bütün V. PH-Responsive Polymers. Polym. Chem. 2017, 8 (1), 144–176. 10.1039/C6PY01872F.

[ref5] Deirram N.; Zhang C.; Kermaniyan S. S.; Johnston A. P. R.; Such G. K. PH-Responsive Polymer Nanoparticles for Drug Delivery. Macromol. Rapid Commun. 2019, 40 (10), 1800917 10.1002/marc.201800917. 30835923

[ref6] Zhang D.; Wang L.; Zhang X.; Bao D.; Zhao Y. Polymeric Micelles for PH-Responsive Lutein Delivery. J. Drug Delivery Sci. Technol. 2018, 45, 281–286. 10.1016/j.jddst.2018.03.023.

[ref7] Suedee R.; Jantarat C.; Lindner W.; Viernstein H.; Songkro S.; Srichana T. Development of a PH-Responsive Drug Delivery System for Enantioselective-Controlled Delivery of Racemic Drugs. J. Controlled Release 2010, 142 (1), 122–131. 10.1016/j.jconrel.2009.10.011. 19857533

[ref8] Ding H.; Tan P.; Fu S.; Tian X.; Zhang H.; Ma X.; Gu Z.; Luo K. Preparation and Application of pH-Responsive Drug Delivery Systems. J. Controlled Release 2022, 348, 206–238. 10.1016/j.jconrel.2022.05.056. 35660634

[ref9] Horcajada P.; Serre C.; Vallet-Regí M.; Sebban M.; Taulelle F.; Férey G. Metal-Organic Frameworks as Efficient Materials for Drug Delivery. Angew. Chem., Int. Ed. Engl. 2006, 45 (36), 5974–5978. 10.1002/anie.200601878. 16897793

[ref10] Horcajada P.; Chalati T.; Serre C.; Gillet B.; Sebrie C.; Baati T.; Eubank J. F.; Heurtaux D.; Clayette P.; Kreuz C.; et al. Porous Metal-Organic-Framework Nanoscale Carriers as a Potential Platform for Drug Delivery and Imaging. Nat. Mater. 2010, 9 (2), 172–178. 10.1038/nmat2608. 20010827

[ref11] Chowdhury M. A. Metal-Organic-Frameworks for Biomedical Applications in Drug Delivery, and as MRI Contrast Agents. J. Biomed. Mater. Res. Part A 2017, 105 (4), 1184–1194. 10.1002/jbm.a.35995. 28033653

[ref12] Della Rocca J.; Liu D.; Lin W. Nanoscale Metal-Organic Frameworks for Biomedical Imaging and Drug Delivery. Acc. Chem. Res. 2011, 44 (10), 957–968. 10.1021/ar200028a. 21648429 PMC3777245

[ref13] Howarth A. J.; Liu Y.; Li P.; Li Z.; Wang T. C.; Hupp J. T.; Farha O. K. Chemical, Thermal and Mechanical Stabilities of Metal–Organic Frameworks. Nat. Rev. Mater. 2016, 1 (3), 15018 10.1038/natrevmats.2015.18.

[ref14] Yaghi O. M.; Li H. Hydrothermal Synthesis of a Metal-Organic Framework Containing Large Rectangular Channels. J. Am. Chem. Soc. 1995, 117 (41), 10401–10402. 10.1021/ja00146a033.

[ref15] Yaghi O. M.; Li G.; Li H. Selective Binding and Removal of Guests in a Microporous Metal–Organic Framework. Nature 1995, 378 (6558), 703–706. 10.1038/378703a0.

[ref16] Wang W.; Yu Y.; Jin Y.; Liu X.; Shang M.; Zheng X.; Liu T.; Xie Z. Two-Dimensional Metal-Organic Frameworks: From Synthesis to Bioapplications. J. Nanobiotechnology 2022, 20 (1), 1–17. 10.1186/s12951-022-01395-9. 35501794 PMC9059454

[ref17] Horcajada P.; Serre C.; McKinlay A. C.; Morris R. E. Biomedical Applications of Metal–Organic Frameworks. Metal-Organic Framework: Applications from Catalysis to Gas Storage 2011, 213–250. 10.1002/9783527635856.ch10.

[ref18] Lawson H. D.; Walton S. P.; Chan C. Metal-Organic Frameworks for Drug Delivery: A Design Perspective. ACS Appl. Mater. Interfaces 2021, 13 (6), 7004–7020. 10.1021/acsami.1c01089. 33554591 PMC11790311

[ref19] Spokoyny A. M.; Kim D.; Sumrein A.; Mirkin C. A. Infinite Coordination Polymer Nano- and Microparticle Structures. Chem. Soc. Rev. 2009, 38 (5), 1218–1227. 10.1039/b807085g. 19384433

[ref20] Lu W.; Wei Z.; Gu Z.-Y.; Liu T.-F.; Park J.; Park J.; Tian J.; Zhang M.; Zhang Q.; Gentle T. III; et al. Tuning the Structure and Function of Metal–Organic Frameworks via Linker Design. Chem. Soc. Rev. 2014, 43 (16), 5561–5593. 10.1039/C4CS00003J. 24604071

[ref21] Spokoyny A. M.; Kim D.; Sumrein A.; Mirkin C. A. Infinite Coordination Polymer Nano- and Microparticle Structures. Chem. Soc. Rev. 2009, 38 (5), 1218 10.1039/b807085g. 19384433

[ref22] Wang Y.; Yan J.; Wen N.; Xiong H.; Cai S.; He Q.; Hu Y.; Peng D.; Liu Z.; Liu Y. Metal-Organic Frameworks for Stimuli-Responsive Drug Delivery. Biomaterials 2020, 230, 119619 10.1016/j.biomaterials.2019.119619. 31757529

[ref23] Zhang J.; He M.; Nie C.; He M.; Pan Q.; Liu C.; Hu Y.; Yi J.; Chen T.; Chu X. Biomineralized Metal–Organic Framework Nanoparticles Enable Enzymatic Rolling Circle Amplification in Living Cells for Ultrasensitive MicroRNA Imaging. Anal. Chem. 2019, 91 (14), 9049–9057. 10.1021/acs.analchem.9b01343. 31274280

[ref24] Zhou C.; Yang Q.; Zhou X.; Jia N. PDA-Coated CPT@MIL-53(Fe)-Based Theranostic Nanoplatform for pH-Responsive and MRI-Guided Chemotherapy. J. Mater. Chem. B 2022, 10 (11), 1821 10.1039/D1TB02339J. 35201249

[ref25] Chen W.; Habraken T. C. J.; Hennink W. E.; Kok R. J. Polymer-Free Drug-Eluting Stents: An Overview of Coating Strategies and Comparison with Polymer-Coated Drug-Eluting Stents. Bioconjugate Chem. 2015, 26 (7), 1277–1288. 10.1021/acs.bioconjchem.5b00192. 26041505

[ref26] Mévellec V.; Roussel S.; Tessier L.; Chancolon J.; Mayne-L’Hermite M.; Deniau G.; Viel P.; Palacin S. Grafting Polymers on Surfaces: A New Powerful and Versatile Diazonium Salt-Based One-Step Process in Aqueous Media. Chem. Mater. 2007, 19 (25), 6323–6330. 10.1021/cm071371i.

[ref27] Surblé S.; Serre C.; Mellot-Draznieks C.; Millange F.; Férey G. A New Isoreticular Class of Metal-Organic-Frameworks with the MIL-88 Topology. Chem. Commun. 2006, 3, 284–286. 10.1039/B512169H. 16391735

[ref28] Horcajada P.; Salles F.; Wuttke S.; Devic T.; Heurtaux D.; Maurin G.; Vimont A.; Daturi M.; David O.; Magnier E.; et al. How Linker’s Modification Controls Swelling Properties of Highly Flexible Iron(III) Dicarboxylates MIL-88. J. Am. Chem. Soc. 2011, 133 (44), 17839–17847. 10.1021/ja206936e. 21950795

[ref29] Horcajada P.; Serre C.; Maurin G.; Ramsahye N. A.; Balas F.; Vallet-Regí M.; Sebban M.; Taulelle F.; Férey G. Flexible Porous Metal-Organic Frameworks for a Controlled Drug Delivery. J. Am. Chem. Soc. 2008, 130 (21), 6774–6780. 10.1021/ja710973k. 18454528

[ref30] Serre C.; Mellot-Draznieks C.; Surblé S.; Audebrand N.; Filinchuk Y.; Férey G. Role of Solvent-Host Interactions That Lead to Very Large Swelling of Hybrid Frameworks. Science 2007, 315 (5820), 1828–1831. 10.1126/science.1137975. 17395825

[ref31] Pham H.; Ramos K.; Sua A.; Acuna J.; Slowinska K.; Nguyen T.; Bui A.; Weber M. D. R.; Tian F. Tuning Crystal Structures of Iron-Based Metal–Organic Frameworks for Drug Delivery Applications. ACS Omega 2020, 5, 3418–3427. 10.1021/acsomega.9b03696. 32118156 PMC7045591

[ref32] Tamames-Tabar C.; Cunha D.; Imbuluzqueta E.; Ragon F.; Serre C.; Blanco-Prieto M. J.; Horcajada P. Cytotoxicity of Nanoscaled Metal–Organic Frameworks. J. Mater. Chem. B 2014, 2 (3), 262–271. 10.1039/C3TB20832J. 32261505

[ref33] Shekhah O.; Liu J.; Fischer R. A.; Wöll C. MOF Thin Films: Existing and Future Applications. Chem. Soc. Rev. 2011, 40 (2), 1081–1106. 10.1039/c0cs00147c. 21225034

[ref34] Gascon J.; Aguado S.; Kapteijn F. Manufacture of Dense Coatings of Cu_3_(BTC)_2_ (HKUST-1) on α-Alumina. Microporous Mesoporous Mater. 2008, 113 (1–3), 132–138. 10.1016/j.micromeso.2007.11.014.

[ref35] Hermes S.; Schröder F.; Chelmowski R.; Wöll C.; Fischer R. A. Selective Nucleation and Growth of Metal–Organic Open Framework Thin Films on Patterned COOH/CF_3_-Terminated Self-Assembled Monolayers on Au(111). J. Am. Chem. Soc. 2005, 127 (40), 13744–13745. 10.1021/JA053523L. 16201767

[ref36] Biemmi E.; Scherb C.; Bein T. Oriented Growth of the Metal Organic Framework Cu_3_(BTC)_2_(H_2_O)_3_·xH_2_O Tunable with Functionalized Self-Assembled Monolayers. J. Am. Chem. Soc. 2007, 129 (26), 8054–8055. 10.1021/ja0701208. 17552519

[ref37] Scherb C.; Schödel A.; Bein T. Directing the Structure of Metal-Organic Frameworks by Oriented Surface Growth on an Organic Monolayer. Angew. Chem., Int. Ed. Engl. 2008, 47 (31), 5777–5779. 10.1002/anie.200704034. 18604800

[ref38] Bui A.; Guillen S. G.; Sua A.; Nguyen T. C.; Ruiz A.; Carachure L.; Weber M. D. R.; Cortez A.; Tian F. Iron-Containing Metal-Organic Framework Thin Film as a Drug Delivery System. Colloids Surfaces A Physicochem. Eng. Asp. 2022, 650, 129611 10.1016/j.colsurfa.2022.129611. PMC9289567 35860194

[ref39] Zacher D.; Shekhah O.; Wöll C.; Fischer R. A. Thin Films of Metal–Organic Frameworks. Chem. Soc. Rev. 2009, 38 (5), 1418–1429. 10.1039/b805038b. 19384445

[ref40] Stavila V.; Volponi J.; Katzenmeyer A. M.; Dixon M. C.; Allendorf M. D. Kinetics and Mechanism of Metal–Organic Framework Thin Film Growth: Systematic Investigation of HKUST-1 Deposition on QCM Electrodes. Chem. Sci. 2012, 3 (5), 1531 10.1039/c2sc20065a.

[ref41] Biemmi E.; Darga A.; Stock N.; Bein T. Direct Growth of Cu_3_(BTC)_2_(H_2_O)_3_·xH_2_O Thin Films on Modified QCM-Gold Electrodes – Water Sorption Isotherms. Microporous Mesoporous Mater. 2008, 114 (1), 380–386. 10.1016/j.micromeso.2008.01.024.

[ref42] Heinke L.; Tu M.; Wannapaiboon S.; Fischer R. A.; Wöll C. Surface-Mounted Metal-Organic Frameworks for Applications in Sensing and Separation. Microporous Mesoporous Mater. 2015, 216, 200–215. 10.1016/j.micromeso.2015.03.018.

[ref43] Shekhah O.; Hirai K.; Wang H.; Uehara H.; Kondo M.; Diring S.; Zacher D.; Fischer R. A.; Sakata O.; Kitagawa S.; et al. MOF-on-MOF Heteroepitaxy: Perfectly Oriented [Zn_2_(Ndc)_2_(Dabco)]_n_ Grown on [Cu_2_(Ndc)_2_(Dabco)]_n_ Thin Films. Dalt. Trans. 2011, 40 (18), 4954 10.1039/c0dt01818j. 21437299

[ref44] Zhou X. L.; Yang Y.; Wang S.; Liu X. W. Surface Plasmon Resonance Microscopy: From Single-Molecule Sensing to Single-Cell Imaging. Angew. Chemie Int. Ed. 2020, 59 (5), 1776–1785. 10.1002/anie.201908806. PMC7020607 31531917

[ref45] Yu H.; Shan X.; Wang S.; Tao N. Achieving High Spatial Resolution Surface Plasmon Resonance Microscopy with Image Reconstruction. Anal. Chem. 2017, 89 (5), 2704–2707. 10.1021/acs.analchem.6b05049. 28194944

[ref46] Garcia A.; Wang K.; Bedier F.; Benavides M.; Wan Z.; Wang S.; Wang Y. Plasmonic Imaging of Electrochemical Reactions at Individual Prussian Blue Nanoparticles. Front. Chem. 2021, 9, 723 10.3389/fchem.2021.718666. PMC8450507 34552911

[ref47] Wang W.; Yang Y.; Wang S.; Nagaraj V. J.; Liu Q.; Wu J.; Tao N. Label-Free Measuring and Mapping of Binding Kinetics of Membrane Proteins in Single Living Cells. Nat. Chem. 2012, 4 (10), 846–853. 10.1038/nchem.1434. 23000999 PMC3660014

[ref48] Sauerbrey G. Verwendung von Schwingquarzen Zur Wägung Dünner Schichten Und Zur Mikrowägung. Zeitschrift für Phys. 1959, 155 (2), 206–222. 10.1007/BF01337937.

[ref49] Ma M.; Noei H.; Mienert B.; Niesel J.; Bill E.; Muhler M.; Fischer R. A.; Wang Y.; Schatzschneider U.; Metzler-Nolte N. Iron Metal-Organic Frameworks MIL-88B and NH_2_ -MIL-88B for the Loading and Delivery of the Gasotransmitter Carbon Monoxide.. Chem. - A Eur. J. 2013, 19 (21), 6785–6790. 10.1002/chem.201201743. 23536364

[ref50] Ma M.; Bétard A.; Weber I.; Al-Hokbany N. S.; Fischer R. A.; Metzler-Nolte N. Iron-Based Metal-Organic Frameworks MIL-88B and NH_2_-MIL-88B: High Quality Microwave Synthesis and Solvent-Induced Lattice “Breathing.. Cryst. Growth Des. 2013, 13 (6), 2286–2291. 10.1021/cg301738p.

[ref51] Hoshi A.; Yanai R.; Kuretani K. Toxicity of Terephthalic Acid. Chem. Pharm. Bull. 1968, 16 (9), 1655–1660. 10.1248/cpb.16.1655. 5709237

[ref52] Institute of Medicine. Dietary Reference Intakes for Vitamin A, Vitamin K, Arsenic, Boron, Chromium, Copper, Iodine, Iron, Manganese, Molybdenum, Nickel, Silicon, Vanadium, and Zinc; The National Academies Press, 2001. 10.17226/10026. 25057538

