# Supplementary material for: pH-Responsive Metal–Organic Framework Thin Film for Drug Delivery
Source: Langmuir. 2022 Dec 14;38(51):16014–23. doi: 10.1021/acs.langmuir.2c02497 (PMC9798862; doi:10.1021/acs.langmuir.2c02497)
Supplement: Supplementary file 1 — la2c02497_si_001.pdf [file la2c02497_si_001.pdf]

pH-Responsive Metal-Organic Framework Thin Film for Drug Delivery

Steven G. Guillen[1Δ], Jacob Parres-Gold[2Φ], Angel Ruiz[1Γ], Ethan Lucsik[1], Benjamin Dao[1], Tran K. L. Hang[1], Megan Chang[2Λ], Adaly O. Garcia[2], Yixian Wang[2] and Fangyuan Tian[1]**

1. Department of Chemistry and Biochemistry, California State University Long Beach, 1250 Bellflower Blvd., Long Beach, CA 90840

2. Department of Chemistry and Biochemistry, California State University Los Angeles, 5151 State University Dr., Los Angeles, CA 90032

Supporting Information

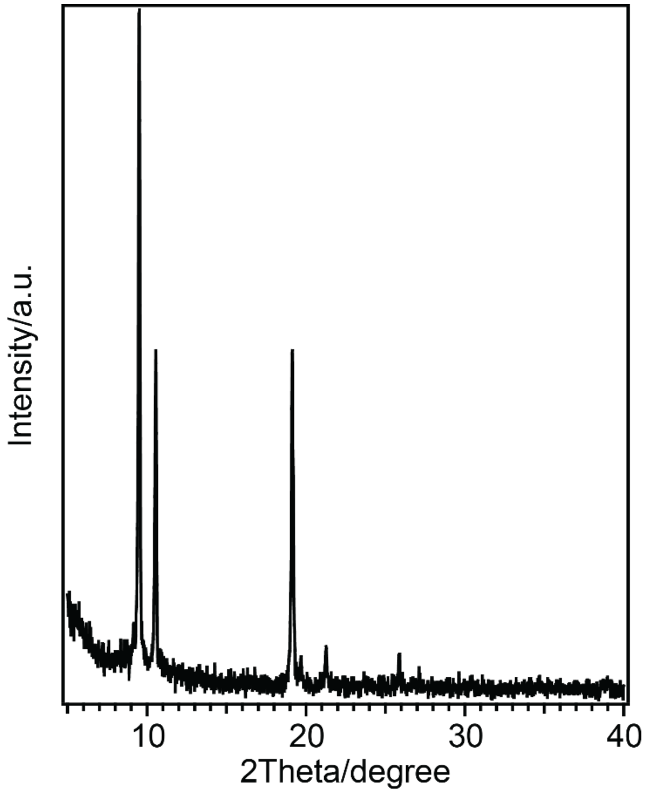

Figure S1. Powder X-ray diffraction pattern for MIL-88B(Fe) extracted from the synthesis mother solution after solvent exchange.

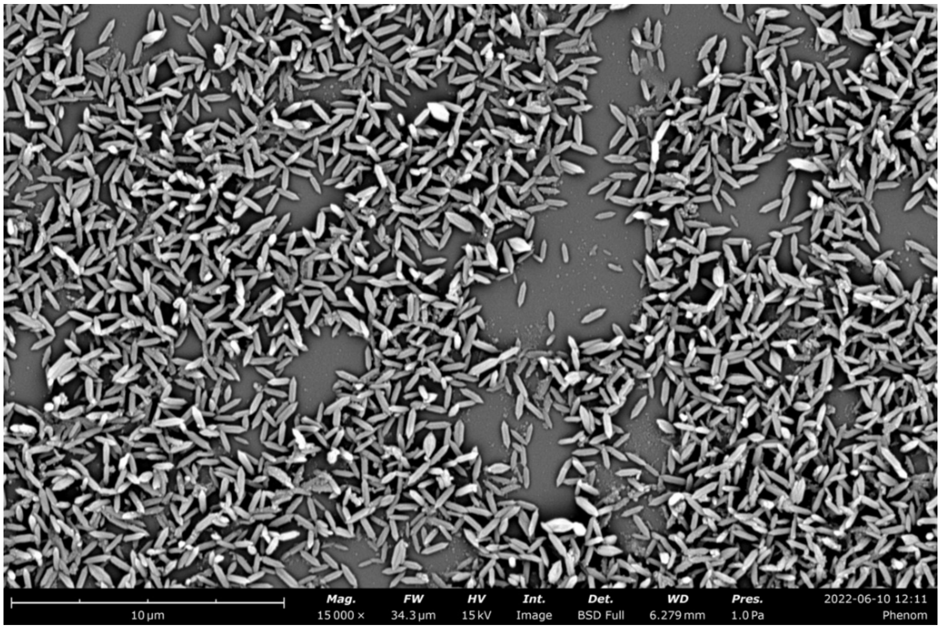

Figure S2. SEM image of MIL-88B(Fe) after synthesis.
